# How to reduce the number of children awaiting the diagnosis of intellectual disability in Brazil?

**DOI:** 10.1192/j.eurpsy.2021.1913

**Published:** 2021-08-13

**Authors:** T. Tafla, D. Brunoni, L.R. Carreiro, A. Seabra, L. Da Silva, A.C. Rossi, P.H. Dos Santos, M.C. Teixeira

**Affiliations:** 1 Postgraduate Program In Developmental Disorders, Mackenzie Presbyterian University, São Paulo, Brazil; 2 Postgraduate Program In Electrical And Computer Engineering, Mackenzie Presbyterian University, São Paulo, Brazil

**Keywords:** Decision-making Process, intellectual disability, Business Process Management System, Data Analytics

## Abstract

**Introduction:**

In Brazil there is high number of children with Intellectual Disability (ID) who begin basic education but did not receive a diagnosis. The basic education teachers can be important agents in identifying signs of ID in the student so that they can be referred to health services.

**Objectives:**

To develop and implement a decision-making model for basic education teachers to identify students with predictive signs of ID.

**Methods:**

The sample was composed by 51 teachers from 20 public schools and their 1758 students eligible for the study enrolled in a educational network in São Paulo state, Brazil. A standardized model was developed for the evaluation process using an open-source software named BONITA. For the screening of students with ID signs the teachers answered a checklist based on the diagnostic criteria of the DSM-5 and the students were evaluated with neuropsychological test WASI (Wechsler Abbreviated Scale of Intelligence) and neuropsychiatric assessment. A Classification Based on Association Rules (CBA) generated the predictive models of sensitivity for confirming ID from the items in the checklists.

**Results:**

35 children had suspected ID. The CBA showed an accuracy of 82%, identifying only 1 false-negative case and 3 false-positive cases for ID. According to the teachers, the most accurate signs were deficits in abstract thinking skills, deficits in communication and conversation and difficulties in emotional regulation in social interactions.

**Conclusions:**

The decision-making model by elementary school teachers to identify students with ID showed high levels of sensitivity and can help the waiting for diagnosis.

**Disclosure:**

No significant relationships.

